# A Systematic Review and Meta‐Analysis on the Levels of Supportive Care Needs in Adults With Cancer

**DOI:** 10.1002/pon.70477

**Published:** 2026-04-29

**Authors:** Caroline B. Silveira, Anderson Rech, Maria Petropoulou, Pietro S. Rössler, Francine F. de Souza, Helena B. Böhmer, Amanda L. Giacomet, Richard Alejandro B. de Barros, Lucélia de C. de Barros, Vagner R. Z. B. Pereira, Nanci da S. T. Junqueira, Pedro Lopez

**Affiliations:** ^1^ Grupo de Pesquisa Em Exercício Para Populações Clínicas (GPCLIN) Universidade de Caxias do Sul Caxias do Sul Brazil; ^2^ Programa de Pós‐Graduação em Ciências da Saúde Universidade de Caxias do Sul Caxias do Sul Brazil; ^3^ Curso de Educação Física Universidade de Caxias do Sul Caxias do Sul Brazil; ^4^ Institute of Medical Biometry and Statistics Faculty of Medicine and Medical Center University of Freiburg Freiburg Germany; ^5^ Curso de Medicina Universidade de Caxias do Sul Caxias do Sul Brazil; ^6^ Curso de Biomedicina Universidade de Caxias do Sul Caxias do Sul Brazil; ^7^ Hospital Geral de Caxias do Sul Fundação da Universidade de Caxias do Sul Caxias do Sul Brazil; ^8^ Institute Santé Hospital Regional Terezinha Gaio Basso São Miguel do Oeste Brazil; ^9^ School of Medical and Health Sciences Edith Cowan University Joondalup Australia; ^10^ Pleural Medicine Unit Institute for Respiratory Health Perth Australia

**Keywords:** cancer, determinants, neoplasm, oncology, unmet needs

## Abstract

**Objective:**

To estimate the levels of supportive care needs among adults with cancer using the Supportive Care Needs Survey–Short Form 34 (SCNS‐SF34) and to examine demographic, clinical, and treatment‐related determinants.

**Methods:**

A systematic review and meta‐analysis was conducted (CRD42024604977). Studies reporting SCNS‐SF34 scores in adults with cancer were included. Searches were performed across six different databases (November 2024; updated August 2025). Random‐effects meta‐analyses were undertaken to estimate the pooled mean scores across the five SCNS‐SF34 domains and total score. Subgroup and meta‐regression analyses explored potential determinants.

**Results:**

Ninety‐five studies (*n* = 32,493) were analysed. The unmet needs observed tended to be higher in the *Health systems and information* (Mean estimate [95% CI] = 38.4 [34.6–42.2]) and *Psychological* (Mean estimate [95% CI] = 35.9 [32.5–39.3]) domains, followed by *Physical and daily living* (Mean estimate [95% CI] = 31.2 [27.7–34.6]) and *Patient care and* *support* (Mean estimate [95% CI] = 30.0 [26.2–33.8]). The lowest needs were reported in the *Sexuality* domain (Mean estimate [95% CI] = 21.3 [18.1–24.5]). Cancer type influenced all domains, except for the psychological domain. Treatment status only influenced the health system and information domain. Older age was consistently associated with lower unmet needs in the health system and information, psychological and patient care and support domains, while surgical treatment was associated with lower needs in physical, patient care, and sexuality domains.

**Conclusions:**

The level of supportive care needs was identified, with informational and psychological domains representing the greatest burden. These needs vary according to cancer type, age, and treatment characteristics, supporting the need for tailored, multidisciplinary strategies to address unmet needs across the cancer continuum.

## 1 Background

Cancer represents the second leading chronic non‐communicable disease worldwide [1], accounting for a substantial proportion of global mortality and premature adult deaths [2]. A cancer diagnosis is frequently associated with life‐course disruption [3] and psychological distress during treatment [4], which may persist for years after its completion. As cancer survival continues to improve, an increasing number of individuals will experience treatment‐related side effects and live with the long‐term consequences of the disease, underscoring the need for enhanced and sustainable supportive care for this population.

In oncology, supportive care refers to a person‐centred approach that addresses the informational, emotional, social, and physical needs of patients with cancer across the disease trajectory [5]. This model reflects an evolving understanding of cancer as a condition with meaningful psychosocial implications, extending beyond disease control and survival alone. For example, individuals may experience symptoms such as fatigue and pain [6, 7], alongside emotional disturbances including anxiety, depressed mood, and a sense of personal crisis or life disruption commencing at the diagnosis [8]. During active treatment, surgery [9], chemotherapy [10], radiotherapy [11] and immunotherapy [12] are associated with a broad range of adverse effects that can further impair physical functioning and psychological well‐being. Following completion of curative treatment, many patients face ongoing uncertainty, persistent symptoms, and cancer‐related comorbidities, contributing to long‐term distress [13, 14]. In advanced stages, cancer is frequently accompanied by profound physical, emotional, and existential suffering [15]. Therefore, understanding and systematically assessing patients' unmet needs across the cancer continuum is critical for addressing the persistent psychosocial burden associated with the disease and significantly increase cancer care.

Previous systematic reviews [16, 17, 18] have sought to identify and synthesise areas in which patients require supportive care across the cancer care continuum. However, these reviews were predominantly narrative in nature and lacked quantitative synthesis, limiting the ability to estimate the levels and variability of supportive care needs across domains and patient populations. Moreover, demographic, clinical, and treatment‐related factors are likely to influence how individuals experience and navigate the cancer trajectory, underscoring the need for a comprehensive examination of determinants of unmet needs. Therefore, this systematic review and meta‐analysis aimed to estimate the levels of supportive care needs among patients with cancer and to explore potential demographic, clinical, and treatment‐related determinants associated with these needs. This review focused on the Supportive Care Needs Survey–Short Form 34 (SCNS‐SF34) [19], one of the most widely used instruments for assessing supportive care needs in oncology, encompassing domains related to health system and information, psychological needs, patient care and support, physical and daily living needs, and sexuality. Such evidence is essential to inform clinical practice, support multidisciplinary care planning, and guide the development of targeted, patient‐centred supportive care interventions.

## 2 Methods

### 2.1 Search Strategy and Study Selection Procedure

This systematic review and meta‐analysis examined the levels of supportive care needs using the SCNS‐SF34 [19] in adult patients with cancer. All procedures and reporting followed the *Cochrane Back Review Group* [20] and the Preferred Reporting Items for Systematic Reviews and Meta‐Analyses (PRISMA) statement [21, 22]. This systematic review was prospectively registered in the International Prospective Register of Systematic Reviews (PROSPERO: CRD42024604977).

Cross‐sectional or longitudinal studies presenting baseline data on SCNS‐SF34 domains, including the *Health systems and information*, *Psychological*, *Patient care and* *support*, *Physical and daily living* and *Sexuality* needs domains, in adult patients (≥ 18 years) with any cancer diagnosis were eligible. When longitudinal studies were included, only baseline measurements were considered to ensure independence of observations and comparability across studies. Publications in English, Spanish, and Portuguese were included, with no restriction on publication year. Articles were excluded when not reporting sufficient information (mean and standard deviation [SD], standard error [SE] or 95% confidence intervals [95% CI], or median and interquartile ranges [IQR]) on the variables of interest.

A comprehensive systematic search was conducted in PubMed, CINAHL (via EBSCOhost), EMBASE, Web of Science, LILACS, and SciELO databases. The systematic search was conducted in November 2024, with no date restrictions, and subsequently updated in August 2025. The search strategy was developed to identify studies investigating supportive care needs in adult patients with cancer. In addition to the electronic search, a manual search was carried out in the reference lists of included studies and previously published systematic reviews on the same topic (i.e., *backward citation chasing*), and articles citing the validation study of the SCNS‐SF34 (i.e., *forward citation chasing*).

Study selection was conducted in a two‐stage process in accordance with PRISMA recommendations. Initially, pairs of five independent researchers (CBS, PSR, FFS, HBB, ALG) screened titles and abstracts based on the inclusion criteria. Full‐text articles of potentially eligible studies were then independently assessed to confirm inclusion. Disagreements were resolved by consensus or a third researcher (PL).

### 2.2 Data Extraction

Data extraction was performed by five independent researchers (CBS, PSR, FFS, HBB, ALG) and checked by another researcher (PL) using a standardised form. Extracted variables included study characteristics, participant characteristics (age [continuous], sex [proportion of males]), cancer type [breast and gynaecologic; gastrointestinal; head, neck, and endocrine cancers; haematologic; neurological and brain; rare and miscellaneous; thoracic; urogenital; and mixed cancers] and stage [proportion of stage III‐IV; proportion of patients with metastasis], and treatment types [proportion of patients with surgery, radiotherapy, chemotherapy, and/or hormone therapy]. Regarding the SCNS‐SF34 domains, these were extracted in their standardised (range: 0–100) or summed score, extracting mean, SD, SE and/or 95% CI, or median and IQR. When studies reported only medians and dispersion measures (IQR or range), these values were converted into mean and SD using established methods [23].

### 2.3 Data Analysis

A random‐effects meta‐analysis [24] was undertaken to estimate the mean and 95% CI of *Health systems and information*, *Psychological*, *Patient care and* *support*, *Physical and daily living* and *Sexuality* domains as well as the overall score of the SCNS‐SF34. Given the expected clinical and methodological heterogeneity across studies, random‐effects models were used to provide more conservative pooled estimates. Higher scores indicated higher needs of supportive care in their respective domains. Statistical heterogeneity was assessed using the Cochran's Q test and estimated by I^2^ statistic and the between‐study variance (${\hat{\tau }}^{2}$), with a substantial heterogeneity defined by a threshold *p*‐value of 0.1 or I^2^ values greater than 50%.

Moreover, the potential sources of heterogeneity (i.e., outliers) between studies was explored by omitting one study at a time (i.e., leave‐one‐out method) and comparing the resulting pooled estimates with the original estimates, whereas the presence of publication bias was assessed using contour‐enhanced funnel plots and Egger's test [25], with a *p*‐value < 0.1 considered indicative of publication bias. When necessary, the trim‐and‐fill computation was used to estimate the effect of publication bias on the interpretation of results. Subgroup and meta‐regression analyses were conducted to examine the influence of demographic variables (age, sex) and clinical variables (cancer type, stage, treatment status and treatment type) on the outcomes. Analyses were conducted using the package ‘meta’ [26] and ‘metafor’ [27] from R (R Core Team, 2020).

## 3 Results

A total of 5094 records were identified through databases search, with 3737 records remaining after duplicate removal. After screening titles and abstracts, 2730 records were excluded as irrelevant to the research question, resulting in 1007 records eligible for full‐text review. A total of 924 reports met the exclusion criteria, while 12 additional studies [28, 29, 30, 31, 32, 33, 34, 35, 36, 37, 38, 39] were identified through updated search. Finally, 95 articles [19, 28, 29, 30, 31, 32, 33, 34, 35, 36, 37, 38, 39, 40, 41, 42, 43, 44, 45, 46, 47, 48, 49, 50, 51, 52, 53, 54, 55, 56, 57, 58, 59, 60, 61, 62, 63, 64, 65, 66, 67, 68, 69, 70, 71, 72, 73, 74, 75, 76, 77, 78, 79, 80, 81, 82, 83, 84, 85, 86, 87, 88, 89, 90, 91, 92, 93, 94, 95, 96, 97, 98, 99, 100, 101, 102, 103, 104, 105, 106, 107, 108, 109, 110, 111, 112, 113, 114, 115, 116, 117, 118, 119, 120, 121] were included in this systematic review and meta‐analysis. The selection process is illustrated in Supporting Information S1: Figure S1.

### 3.1 Participant Characteristics

A total of 32,493 patients with cancer (34.4% male) were included in the analysed studies, with most studies conducted in Asia (*n* = 38, 19.5%), Europe (*n* = 24, 12.3%) and North America (*n* = 17, 8.7%). The median age was 55.3 years (interquartile range [IQR], 52.8–60.9). The majority of patients had breast or gynaecological cancers (30.5%), followed by gastrointestinal (12.6%), head, neck and endocrine (9.5%), prostate (6.3%), lung (4.2%), neurological (4.2%), and haematological cancers (3.2%). In addition, 29.5% of studies included patients with multiple cancer types. Few studies reported cancer stage, with only 17.8% of patients described as having advanced disease and 7.1% as having metastatic disease. Regarding treatments, surgery was reported in 29.7% of patients, followed by chemotherapy (29.0%), radiotherapy (20.5%) and hormone therapy (7.2%). Regarding the study design, most studies were cross‐sectional (*n* = 73, 37.4%). Characteristics of the included studies are presented in Supporting Information S1: Table S1.

### 3.2 Health Systems and Information Domain

Seventy studies were included in the meta‐analysis for the *Health systems and information* domain. The mean score was estimated at 38.4 (95% CI: 34.6–42.2; *p* < 0.001; Table 1). The between‐study variance ${\hat{\tau }}^{2}$ was 255.7 (I^2^ = 99.7%). Egger's test indicated the presence of funnel plot asymmetry (*p* = 0.015; Supporting Information S1: Figure S2). The questionnaire‐based sum score (0–40) was reported by 17 studies and estimated at 27.1 (95% CI: 23.9–30.3; Supporting Information S1: Table S2).

**TABLE 1:** Overall and cancer‐type subgroup mean scores (95% CI) for the *Health systems and information* domain.

	k	Mean	95% CI	*p*‐value	Q	I^2^	${\hat{\tau }}^{2}$	*p*‐value
Overall								
Mean estimate	70	38.4	34.6 to 42.2	< 0.001	20,032.7	99.7%	255.7	< 0.001
Cancer type								
Breast and gynaecologic	19	44.8	36.3 to 53.3	< 0.001	1570.5	98.9%	350.4	< 0.001
Gastrointestinal	10	35.0	26.6 to 43.4	< 0.001	577.3	98.4%	181.9	< 0.001
Head, neck, and endocrine	5	31.3	12.8 to 49.8	< 0.001	2073.2	99.8%	445.4	< 0.001
Haematologic	3	34.9	18.6 to 51.1	< 0.001	150.3	98.7%	203.7	< 0.001
Neurological and brain	3	32.5	22.0 to 43.0	< 0.001	29.4	93.2%	79.1	< 0.001
Rare and miscellaneous	2	26.7	21.8 to 31.7	< 0.001	2.8	64.2%	8.2	0.094
Thoracic	2	41.3	16.2 to 66.4	0.001	60.0	98.3%	322.1	< 0.001
Urogenital	4	36.5	9.5 to 63.6	0.082	1348.9	99.8%	760.5	< 0.001
Treatment status								
Current treatment	21	43.9	37.3 to 50.4	< 0.001	2323.0	99.1%	233.3	< 0.001
Previous treatment	24	34.3	27.8 to 40.8	< 0.001	12,987.9	99.8%	262.0	< 0.001

Abbreviations: 95% CI, 95% confidence interval; I^2^, percentage of variation across studies that is due to heterogeneity; k, number of studies; *Q*, Cochran's Q test of heterogeneity.

Differences were observed across cancer types (*Q* = 17.8, *p* = 0.022). The mean score estimation ranged from 26.7 to 44.8, with breast and gynaecological cancers (Mean estimate [95% CI] = 44.8 [36.3–53.3] and thoracic cancers (Mean estimate [95% CI] = 41.3 [16.2–66.4]) exhibiting the highest scores, whereas the miscellaneous tumours (Mean estimate [95% CI] = 26.7 [21.8–31.7]) and head, neck, and endocrine cancers (Mean estimate [95% CI] = 31.3 [12.8–49.8]) exhibited the lowest scores. Differences were also observed regarding the treatment status (*Q* = 4.1, *p* = 0.043), with higher scores estimated for patients during treatment (Mean estimate [95% CI] = 43.9 [37.3–50.4]) than those who received previous treatment (Mean estimate [95% CI] = 34.3 [27.8–40.8]).

In the meta‐regression analysis (Supporting Information S1: Table S3), age (B ± SE = −0.75 ± 0.28, *p* = 0.008) was associated with lower scores in the *Health systems and information* domain. No associations with changes in the score were observed (*p* = 0.140–0.907) for the remaining variables (proportion of males, proportion of patients with stage III–IV disease, proportion of patients with metastasis, and proportion of patients receiving surgery, radiotherapy, chemotherapy, or hormone therapy).

### 3.3 Psychological Domain

Seventy studies were included in the meta‐analysis for the *Psychological* domain. The mean score was estimated at 35.9 (95% CI: 32.5–39.3; *p* < 0.001; Table 2). The between‐study variance ${\hat{\tau }}^{2}$ was 208.5 (I^2^ = 99.5%), with no presence of funnel plot asymmetry (*p* = 0.780; Supporting Information S1: Figure S3). The questionnaire‐based sum score [10, 11, 12, 13, 14, 15, 16, 17, 18, 19, 20, 21, 22, 23, 24, 25, 26, 27, 28, 29, 30, 31, 32, 33, 34, 35, 36, 37, 38, 39, 40, 41, 42, 43, 44, 45, 46, 47, 48, 49, 50] was reported by 18 studies and estimated at 24.3 (95% CI: 21.2–27.3; Supporting Information S1: Table S2).

**TABLE 2:** Overall and cancer‐type subgroup mean scores (95% CI) for the *Psychological* domain.

	k	Mean	95% CI	*p*‐value	Q	I^2^	${\hat{\tau }}^{2}$	*p*‐value
Overall								
Mean estimate	70	35.9	32.5 to 39.3	< 0.001	13,737.9	99.5%	208.5	< 0.001
Cancer type								
Breast and gynaecologic	19	39.0	30.9 to 47.0	< 0.001	2942.0	99.4%	315.5	< 0.001
Gastrointestinal	10	28.7	22.3 to 35.1	< 0.001	362.1	97.5%	103.3	< 0.001
Head, neck, and endocrine	5	32.2	20.6 to 43.7	< 0.001	312.4	98.7%	166.7	< 0.001
Haematologic	3	30.4	18.9 to 42.0	< 0.001	68.1	97.1%	101.8	< 0.001
Neurological and brain	3	36.2	25.6 to 46.8	< 0.001	20.8	90.4%	78.7	< 0.001
Rare and miscellaneous	2	33.4	28.2 to 38.6	< 0.001	2.3	56.7%	7.9	0.129
Thoracic	2	39.1	32.5 to 45.6	< 0.001	3.5	71.4%	16.0	0.062
Urogenital	4	40.6	18.1 to 63.1	< 0.001	912.8	99.7%	523.2	< 0.001
Treatment status								
Current treatment	21	41.7	34.9 to 48.4	< 0.001	3435.8	99.4%	244.7	< 0.001
Previous treatment	24	33.3	28.0 to 38.6	< 0.001	7146.5	99.7%	171.6	< 0.001

Abbreviations: 95% CI, 95% confidence interval; I^2^, percentage of variation across studies that is due to heterogeneity; k, number of studies; *Q*, Cochran's Q test of heterogeneity.

No differences were observed across cancer types (*Q* = 8.1, *p* = 0.426), with the mean score estimation ranging from 28.7 to 40.6. Likewise, differences were not observed regarding treatment status (*Q* = 3.6, *p* = 0.056). Age was associated with lower scores in the *Psychological* domain (B ± SE = −0.60 ± 0.26, *p* = 0.022), whereas the remaining variables (proportion of males, proportion of patients with stage III‐IV, proportion of patients with metastasis, proportion of patients with surgery, radiotherapy, chemotherapy and hormone treatment) were not associated with changes in the score (*p* = 0.224–0.874; Supporting Information S1: Table S4).

### 3.4 Physical and Daily Living Domain

For the supportive care needs in the *Physical and daily living* domain, 71 studies were included in the meta‐analysis. The mean score was estimated at 31.2 (95% CI: 27.7–34.6; *p* < 0.001; Table 3). The between‐study variance ${\hat{\tau }}^{2}$ was 221.6 (I^2^ = 99.8%). Egger's test did not indicate the presence of funnel plot asymmetry (*p* = 0.146; Supporting Information S1: Figure S4). The questionnaire‐based sum score [5, 6, 7, 8, 9, 10, 11, 12, 13, 14, 15, 16, 17, 18, 19, 20, 21, 22, 23, 24, 25] was reported by 18 studies and estimated at 11.8 (95% CI: 10.3–13.4; Supporting Information S1: Table S2).

**TABLE 3:** Overall and cancer‐type subgroup mean scores (95% CI) for the *Physical and daily living* domain.

	k	Mean	95% CI	*p*‐value	Q	I^2^	${\hat{\tau }}^{2}$	*p*‐value
Overall								
Mean estimate	71	31.2	27.7 to 34.6	< 0.001	28,600.0	99.8%	221.6	< 0.001
Cancer type								
Breast and gynaecologic	19	31.7	24.2 to 39.2	< 0.001	2265.8	99.2%	274.1	< 0.001
Gastrointestinal	10	27.0	20.4 to 33.6	< 0.001	764.5	98.8%	111.0	< 0.001
Head, neck, and endocrine	6	21.8	12.2 to 31.3	< 0.001	578.8	99.1%	139.7	< 0.001
Haematologic	3	25.6	15.8 to 35.4	< 0.001	49.6	96.0%	73.0	< 0.001
Neurological and brain	3	31.4	14.9 to 47.8	< 0.001	43.6	95.4%	201.6	< 0.001
Rare and miscellaneous	2	25.1	21.8 to 28.4	< 0.001	0.2	0.0%	0.0	0.700
Thoracic	2	33.5	30.2 to 36.7	< 0.001	0.4	0.0%	0.0	0.530
Urogenital	4	37.2	12.9 to 61.6	< 0.001	754.1	99.6%	615.8	< 0.001
Treatment status								
Current treatment	21	35.4	29.3 to 41.5	< 0.001	2760.9	99.3%	201.9	< 0.001
Previous treatment	25	27.9	22.3 to 33.5	< 0.001	16,795.8	99.9%	199.2	< 0.001

Abbreviations: 95% CI, 95% confidence interval; I^2^, percentage of variation across studies that is due to heterogeneity; k, number of studies; *Q*, Cochran's Q test of heterogeneity.

Differences were observed across cancer types (*Q* = 19.6, *p* = 0.012). The mean score estimation ranged from 21.8 to 37.2. The highest score was observed in studies involving urogenital (Mean estimate [95% CI] = 37.2 [12.9–61.6]) and mixed cancers (Mean estimate [95% CI] = 35.7 [29.0–42.5]), while the lowest scores were observed in studies involving head, neck, and endocrine cancers (Mean estimate [95% CI] = 21.8 [12.2–31.3]) and rare and miscellaneous cancers (Mean estimate [95% CI] = 25.1 [21.8–28.4]). Differences were not observed regarding treatment status (*Q* = 3.2, *p* = 0.076).

In the meta‐regression analysis (Supporting Information S1: Table S5), proportion of patients with surgery (B ± SE = −0.15 ± 0.06, *p* = 0.011) was associated with lower scores in *Physical and daily living* domain. No association was observed between changes in *Physical and daily living* domain and remaining variables (age, proportion of males, proportion of patients with stage III‐IV, proportion of patients with metastasis, radiotherapy, chemotherapy and hormone treatment; *p* = 0.086–0.985).

### 3.5 Patient Care and Support Domain

For the *Patient care and* *support* domain, 66 studies were included in the meta‐analysis. The mean score was estimated at 30.0 (95% CI: 26.2–33.8; *p* < 0.001; Table 4). The between‐study variance ${\hat{\tau }}^{2}$ was 247.7 (I^2^ = 99.8%). Egger's test indicated the presence of funnel plot asymmetry (*p* = 0.002; Supporting Information S1: Figure S5). The questionnaire‐based sum score [5, 6, 7, 8, 9, 10, 11, 12, 13, 14, 15, 16, 17, 18, 19, 20, 21, 22, 23, 24, 25] was reported by 17 studies and estimated at 10.8 (95% CI: 9.5–12.0; Supporting Information S1: Table S2).

**TABLE 4:** Overall and cancer‐type subgroup mean scores (95% CI) for the *Patient care and support* domain.

	k	Mean	95% CI	*p*‐value	Q	I^2^	${\hat{\tau }}^{2}$	*p*‐value
Overall								
Mean estimate	66	30.0	26.2 to 33.8	< 0.001	41,425.8	99.8%	247.7	< 0.001
Cancer type								
Breast and gynaecologic	18	34.7	27.6 to 41.8	< 0.001	1190.3	98.6%	229.6	< 0.001
Gastrointestinal	10	24.0	16.3 to 31.8	< 0.001	1045.3	99.1%	154.8	< 0.001
Head, neck, and endocrine	4	23.6	−0.1 to 47.4	0.051	1685.2	99.8%	585.6	< 0.001
Haematologic	2	27.2	13.8 to 40.7	< 0.001	48.8	98.0%	92.1	< 0.001
Neurological and brain	3	19.6	8.2 to 30.9	< 0.001	60.4	96.7%	97.6	< 0.001
Rare and miscellaneous	2	21.8	19.2 to 24.4	< 0.001	0.2	0.0%	0.0	0.679
Thoracic	2	24.4	18.4 to 30.3	< 0.001	3.4	70.9%	13.2	0.064
Urogenital	4	36.4	6.0 to 66.8	0.019	2299.9	99.9%	959.0	< 0.001
Treatment status								
Current treatment	21	33.3	27.0 to 39.5	< 0.001	2275.7	99.1%	211.3	< 0.001
Previous treatment	23	27.2	20.8 to 33.5	< 0.001	28,802.4	99.9%	239.7	< 0.001

Abbreviations: 95% CI, 95% confidence interval; I^2^, percentage of variation across studies that is due to heterogeneity; k, number of studies; *Q*, Cochran's Q test of heterogeneity.

Differences were observed across cancer types (*Q* = 19.2, *p* = 0.014), with the mean score estimation ranging from 19.6 to 36.4. The highest scores were observed in studies involving patients with urogenital cancers (Mean estimate [95% CI] = 36.4 [6.0–66.8]) and breast and gynaecological cancers (Mean estimate [95% CI] = 34.7 [27.6–41.8]), while the lowest scores were in studies involving neurological and brain (Mean estimate [95% CI] = 19.6 [8.2–30.9]) and rare and miscellaneous cancers (Mean estimate [95% CI] = 21.8 [19.2–24.4]). Differences were not observed regarding treatment status (*Q* = 1.8, *p* = 0.179).

In the meta‐regression analysis (Supporting Information S1: Table S6), age (B ± SE = −0.76 ± 0.29, *p* = 0.009) and proportion of patients with surgery (−0.12 ± 0.06, *p* = 0.045) were associated with lower scores in the *Patient care and* *support* domain. The remaining variables (proportion of males, proportion of patients with stage III‐IV, proportion of patients with metastasis, radiotherapy, chemotherapy and hormone treatment) were not associated with changes in the score (*p* = 0.280–0.941).

### 3.6 Sexuality Domain

For the *Sexuality* domain, the mean score was estimated at 21.3 (95% CI: 18.1–24.5; *p* < 0.001; Table 5) after analysing 71 studies. The between‐study variance ${\hat{\tau }}^{2}$ was 188.3 (I^2^ = 99.7%), with presence of funnel plot asymmetry (*p* = 0.096; Supporting Information S1: Figure S6). The questionnaire‐based sum score [3, 4, 5, 6, 7, 8, 9, 10, 11, 12, 13, 14, 15] was reported by 18 studies and estimated at 5.5 (95% CI: 4.6–6.4; Supporting Information S1: Table S2).

**TABLE 5:** Overall and cancer‐type subgroup mean scores (95% CI) for the *Sexuality* domain.

	k	Mean	95% CI	*p*‐value	Q	I^2^	${\hat{\tau }}^{2}$	*p*‐value
Overall								
Mean estimate	71	21.3	18.1 to 24.5	< 0.001	23,147.2	99.7%	188.3	< 0.001
Cancer type								
Breast and gynaecologic	19	21.7	15.6 to 27.7	< 0.001	1999.8	99.1%	177.1	< 0.001
Gastrointestinal	10	9.7	5.6 to 13.7	< 0.001	262.0	96.6%	41.0	< 0.001
Head, neck, and endocrine	5	13.6	7.2 to 20.0	< 0.001	233.7	98.3%	51.0	< 0.001
Haematologic	3	16.7	8.5 to 24.8	< 0.001	44.4	95.5%	49.5	< 0.001
Neurological and brain	3	20.4	7.6 to 33.2	0.002	17.5	88.5%	118.4	< 0.001
Rare and miscellaneous	2	17.3	14.3 to 20.2	< 0.001	0.9	0.0%	0.0	0.340
Thoracic	2	14.0	9.7 to 18.2	< 0.001	2.3	55.9%	5.4	0.132
Urogenital	4	38.4	15.6 to 61.2	< 0.001	627.3	99.5%	539.7	< 0.001
Treatment status								
Current treatment	21	22.9	16.7 to 29.0	< 0.001	3274.1	99.4%	201.5	< 0.001
Previous treatment	25	20.9	16.3 to 25.5	< 0.001	16,710.0	99.9%	134.9	< 0.001

Abbreviations: 95% CI, 95% confidence interval; I^2^, percentage of variation across studies that is due to heterogeneity; k, number of studies; *Q*, Cochran's Q test of heterogeneity.

Differences were observed across cancer types (*Q* = 32.2, *p* < 0.001). The mean score estimation ranged from 9.7 to 38.4, with the lowest and highest score observed in gastrointestinal (Mean estimate [95% CI] = 9.7 [5.6–13.8]) and urogenital cancers (Mean estimate [95% CI] = 38.4 [15.6–61.2]). Differences were not observed regarding treatment status (*Q* = 0.2, *p* = 0.622).

An association was observed between the proportion of patients with surgery and lower values in *Sexuality* domain (B ± SE = −0.11 ± 0.05, *p* = 0.025). No association was observed between changes in *Sexuality* domain and remaining variables (age, proportion of males, proportion of patients with stage III‐IV, proportion of patients with metastasis, radiotherapy, chemotherapy and hormone treatment; *p* = 0.087–0.934; Supporting Information S1: Table S7).

### 3.7 Total Score

For the total supportive care needs score, the mean score was estimated at 44.5 (95% CI: 34.0–55.3; *p* < 0.001; Table 6) after analysing 16 studies. The between‐study variance ${\hat{\tau }}^{2}$ was 475.6 (I^2^ = 99.7%), with no presence of funnel plot asymmetry (*p* = 0.790; Supporting Information S1: Figure S7).

**TABLE 6:** Overall and cancer‐type subgroup mean scores (95% CI) for the total score.

	k	Mean	95% CI	*p*‐value	Q	I^2^	${\hat{\tau }}^{2}$	*p*‐value
Overall								
Mean estimate	16	44.5	33.8 to 55.3	< 0.001	5261.1	99.7%	475.6	< 0.001
Cancer type								
Breast and gynaecologic	5	43.4	29.7 to 57.1	< 0.001	244.0	98.4%	240.1	< 0.001
Gastrointestinal	3	72.4	52.9 to 92.0	< 0.001	546.4	99.6%	292.3	< 0.001
Urogenital	3	29.1	16.8 to 41.5	< 0.001	64.8	96.9%	117.0	< 0.001
Treatment status								
Current treatment	9	47.7	36.8 to 58.6	< 0.001	1247.6	99.4%	273.1	< 0.001
Previous treatment	3	25.7	8.8 to 42.5	< 0.001	201.8	99.0%	218.6	< 0.001

Abbreviations: 95% CI, 95% confidence interval; I^2^, percentage of variation across studies that is due to heterogeneity; k, number of studies; *Q*, Cochran's Q test of heterogeneity.

Differences were observed across cancer types (*Q* = 13.50, *p* < 0.001). The mean score estimation ranged from 29.1 to 72.5, with gastrointestinal cancers exhibiting the highest scores (Mean estimate [95% CI] = 72.5 [52.9–92.0]), whereas urogenital cancers (Mean estimate [95% CI] = 29.2 [16.8–41.5]) exhibited the lowest scores. Differences were also observed regarding the treatment status (*Q* = 4.6, *p* = 0.032), with higher scores estimated for patients during treatment (Mean estimate [95% CI] = 47.7 [36.8–58.6]) than those who received previous treatment (Mean estimate [95% CI] = 25.7 [8.8–42.5]).

In the meta‐regression analysis, no association was observed between age, proportion of males and proportion of patients with chemotherapy with the total supportive care needs score (*p* = 0.082–0.965; Supporting Information S1: Table S8), while the remaining variables were not investigated due to the small number of studies available (*n* < 10).

## 4 Discussion

This systematic review and meta‐analysis aimed to examine the levels and potential determinants of supportive care needs using the SCNS‐SF34 in patients with cancer. While previous studies have narratively described supportive care needs within specific populations or cancer types [16, 17, 18], this meta‐analysis extends existing knowledge by synthesising evidence across diverse settings and identifying consistent patterns across domains, despite substantial heterogeneity. We observed that the *Health systems and information* and *Psychological* domains tended to show higher unmet needs, followed by the *Physical and daily living* and *Patient care and* *support* domains, while the lowest unmet needs were observed for the *Sexuality* domain. Regarding the potential determinants of supportive care needs, cancer type, treatment status, age, and surgery were the only factors associated with variations in scores. This information is necessary to optimise resource allocation, ensuring that oncology services and patient navigation programs are targeted toward the most vulnerable, and to move beyond a purely biomedical focus to a holistic model that anticipates the high burden of unmet needs.

Overall, we observed substantial heterogeneity across the domains of the SCNS‐SF34, likely reflecting important clinical and methodological differences between the included studies. Although variables such as cancer type and treatment status were explored in subgroup analyses, they did not fully explain the observed heterogeneity. Additionally, potentially relevant factors, such as differences in healthcare systems, cultural contexts, and timing of assessment, were not consistently reported and therefore could not be examined. Variations in sampling strategies and reporting practices may have further contributed to the observed variability. Collectively, these factors highlight the complexity of synthesising supportive care needs across diverse populations and contexts, underscoring the need for cautious interpretation of pooled estimates.

The *Health systems and information* domain tended to show higher mean scores compared to other domains, suggesting that patients frequently perceived gaps in the availability, clarity, and promptness of information related to their disease, treatment options, and navigation of the healthcare system. Several studies refer to this as ‘information‐seeking behavior’ [122, 123, 124, 125]. Notably, these values tended to be higher among those undergoing active treatment, when uncertainty is greater and patients are required to make complex decisions regarding their care. While we observed no differences based on sex or stage in the overall cohort, higher scores were noted specifically in patients with breast and gynaecological cancers, possibly due to the complexity and preference‐sensitive nature of treatment decisions [126, 127, 128]. In lung cancer, higher unmet needs may reflect the complexity of care coordination [129] and challenges in communication around prognosis [130]. To bridge these gaps, the implementation of nurse‐led navigation programs and specific communication strategies are essential to reduce disparities in access to information and improve care pathways [131, 132].

Similarly, potentially high burdens were observed in the *Psychological* domain. Elevated psychological needs may reflect the persistent emotional burden of cancer, such as fear of disease progression, anxiety, uncertainty about the future, and general emotional distress [8], which is often insufficiently addressed within standard care models that prioritise physical treatment over psychosocial support [133]. These psychological unmet needs appear to impact healthcare system utilisation [13, 134, 135] as well as treatment decision‐making [13]. We did not observe differences across cancer types, stages, and treatments, suggesting a high burden of distress irrespective of diagnosis and treatment, despite substantial study variation in need levels. This result partially agrees with previous studies indicating consistently high levels of fear of cancer and general distress [13, 14].

Unmet needs in the *Physical and daily living* domain may arise from the cumulative impact of treatment‐related side effects and functional limitations, which can compromise independence and quality of life throughout the disease trajectory [136]. We observed moderate levels of unmet needs in the *Physical and daily living* domain, with the highest scores found among men with prostate cancer. This result may be explained by the range of treatment‐related toxicities experienced during and after the course of therapy [137, 138]. Additionally, our results may indicate that *Physical and daily living* domain levels were not associated with cancer stage or chemotherapy status, despite these factors commonly being assumed to be important drivers of physical symptom burden [139, 140]. The integration of physical exercise interventions into routine care is increasingly prioritised to mitigate these unmet needs; indeed, physically active patients exhibit lower *Physical and daily living* needs [53], and structured exercise has been shown to significantly reduce fatigue and pain [141, 142].

As observed in the *Patient care and* *support* domain, experiences of care can vary substantially across different health systems, institutions, and oncology care models. Unmet needs in *Patient care and* *support* may reflect gaps in care coordination, continuity of care, and the ability of healthcare systems to provide individualised and accessible support services [143]. For example, qualitative evidence in prostate cancer [144] indicates that unmet needs often derive from challenges regarding trust in health‐care professionals, fragmented continuity of care, complex health system navigation, and limited access to psychosocial and psychosexual support. In women with breast cancer, fragmentation of multidisciplinary care [145] and the transitioning from active treatment to survivorship often result in a profound sense of abandonment or loss of the safety net provided by the clinical team [146]. Collectively, these systemic and transitional challenges likely underpin the elevated unmet needs observed among men with prostate cancer and women with breast cancer in our analysis.

Regarding the *Sexuality* domain, the lowest pooled scores were observed, a finding that appears paradoxical given the high prevalence of treatment‐induced sexual dysfunction across cancer types [147, 148, 149, 150]. This discrepancy likely reflects system‐ and patient‐level barriers rather than a true absence of need [151, 152, 153]. Specifically, this underreporting is often driven by embarrassment, stigma, or a patient perception that sexual well‐being is not a legitimate medical concern within the oncology setting. While men with prostate cancer reported comparatively higher needs, likely attributable to the direct physiological impact of surgery and androgen deprivation therapy [150], the overall low scores across the cohort suggest widespread underreporting, particularly given the discordance with established evidence on sexual dysfunction among cancer patients [147, 148, 149, 150].

Regarding the meta‐regression results, older patients tended to report lower unmet needs compared to younger cohorts across the *Health systems and information*, *Psychological* and *Patient care and* *support* domains. These findings may reflect several underlying factors, including the potential underreporting of unmet needs among older individuals [154] and generational differences in expectations, with older patients often prioritising survival outcomes over psychosocial support [155, 156]. The demographic composition of the studies included may have also influenced these findings, as younger age and female sex are typically associated with higher reported needs [157]. However, no association between sex and *Psychological* needs was observed, and interactions between age and sex were not explored. Importantly, these associations may be influenced by residual confounding, as meta‐regression analyses were based on aggregated study‐level data and did not allow adjustment for multiple covariates simultaneously. Therefore, these findings should be interpreted with caution and considered exploratory and hypothesis‐generating rather than indicative of causal relationships.

Surprisingly, we observed that undergoing surgery was associated with lower unmet needs across the *Physical and daily living*, *Patient care and* *support*, and *Sexuality* domains. This association may be partially influenced by selection bias; as patients selected for surgery are typically younger, have better baseline functional status, and present with earlier‐stage disease [158, 159], which may contribute to lower reported needs. On the other hand, surgery is often perceived as a more definitive intervention with a structured recovery pathway, in contrast to the ongoing uncertainty and complexity of systemic therapies. Lower unmet needs in the *Sexuality* domain may also reflect the role of anticipatory guidance, as surgical procedures are usually accompanied by clearer pre‐treatment counselling [151, 152, 153]. Overall, this association should be interpreted cautiously, as it may reflect underlying differences in patient characteristics rather than a direct effect of surgery itself.

### 4.1 Study Limitations

To the best of our knowledge, this study represents the most comprehensive synthesis of supportive care needs in cancer patients to date. Strengths include the substantial sample size (*n* = 32,943 patients across 95 studies) and the robust statistical approach, utilising meta‐analysis to estimate pooled mean levels and subgroup and meta‐regression to identify potential determinants of unmet needs, aiming to explore independent associations with supportive care needs. However, there are a few limitations. First, our analyses were restricted to studies using SCNS‐SF34, which is the most used instrument to identify supportive care needs. While this criterion excluded data derived from other validated instruments (e.g., CaSUN, CNQ, CSNAT, NA‐ACP and others), this methodological decision was necessary to ensure psychometric homogeneity and facilitate a valid quantitative synthesis of pooled mean scores, which would have been compromised by combining heterogeneous measurement scales. Second, the very high heterogeneity across studies (I^2^ = ∼99%) represents an important limitation, reducing confidence in pooled estimates and limiting their generalisability. This reflects the diverse clinical settings, healthcare systems, and cultural contexts from which the data were derived. While we employed random‐effects models to account for this between‐study variance and utilised meta‐regression to explore potential sources, unmeasured confounders inevitably influenced pooled estimates. Therefore, our results should be interpreted as exploratory and indicative of global trends rather than precise predictive values for specific local settings. Third, although all studies used the SCNS‐SF34, differences in factor structures (four vs. five domains) may have led to inconsistencies in how supportive care needs were conceptualised, potentially influencing pooled domain estimates. Nevertheless, domains were mapped to the original SCNS‐SF34 domains where conceptually appropriate. Fourth, given that meta‐regression was conducted using aggregated study‐level data, the results may be influenced by ecological bias and should not be interpreted as reflecting individual‐level relationships. Therefore, our findings should be interpreted as exploratory and hypothesis‐generating rather than causal.

### 4.2 Clinical Implications

Our findings highlight the need to prioritise supportive care as a central component of oncology practice, particularly in the *Health systems and information* and *Psychological* domains, where unmet needs were mainly observed. Structured communication strategies, patient navigation programs [131, 132], and tailored educational resources should be integrated into routine care to improve patients' understanding and engagement in decision‐making [122, 123, 124, 125]. In parallel, systematic screening and management of psychological distress should be embedded across the cancer continuum, given its consistent burden across cancer types and treatment stages.

Moderate unmet needs in the *Physical and daily living* domain reinforce the importance of integrating exercise and rehabilitation strategies into standard care to mitigate treatment‐related side effects and functional decline [53, 141, 142]. Supportive care should also be individualised, considering differences by age, cancer type, and treatment characteristics. Finally, the low reported needs in the *Sexuality* domain likely reflect underreporting [151, 152, 153], underscoring the importance of proactively addressing sexual health in clinical practice to ensure comprehensive, patient‐centred care.

## 5 Conclusions

In conclusion, our study identified the potential levels of supportive care needs in oncology, with the *Health systems and information* and *Psychological* domains as the areas of greatest concern, followed by persistent challenges in *Physical and daily living* and *Patient care and* *support*. Conversely, the paradoxically low scores in the *Sexuality* domain likely reflect barriers to reporting rather than a lack of need. In addition, we identified that these burdens are not consistent across all cancer and clinical characteristics, with cancer type, treatment status, age, and surgical status emerging as potential determinants of variation. Our study contributes to the field by moving beyond descriptive reporting of unmet needs and providing a synthesis of potential patterns that may inform more targeted and context‐sensitive supportive care strategies.

## Author Contributions

*Conception and design*: Caroline B. Silveira and Pedro Lopez. *Acquisition, analysis, or interpretation of data*: Caroline B. Silveira, Anderson Rech, Maria Petropoulou, Pietro S. Rössler, Francine F. de Souza, Helena B. Böhmer, Amanda L. Giacomet, Richard Alejandro B. de Barros, Lucélia de C. de Barros, Vagner R. Z. B. Pereira, Nanci da S. T. Junqueira and Pedro Lopez. *Drafting of the manuscript*: Caroline B. Silveira and Pedro Lopez. *Critical revision of the manuscript for important intellectual content*: Caroline B. Silveira, Anderson Rech, Maria Petropoulou, Pietro S. Rössler, Francine F. de Souza, Helena B. Böhmer, Amanda L. Giacomet, Richard Alejandro B. de Barros, Lucélia de C. de Barros, Vagner R. Z. B. Pereira, Nanci da S. T. Junqueira and Pedro Lopez. All authors read and approved the final version of the article.

## Funding

The authors have nothing to report.

## Ethics Statement

The authors have nothing to report.

## Conflicts of Interest

The authors declare no conflicts of interest.

## Supporting information


Supporting Information S1

## Data Availability

Data used in the present study such as data extraction templates, forms and analysis will be made available upon reasonable request to the corresponding author.
