# Supplementary material for: A Systematic Review and Meta‐Analysis on the Levels of Supportive Care Needs in Adults With Cancer
Source: Psychooncology. 2026 Apr 29;35:e70477. doi: 10.1002/pon.70477 (PMC13127244; doi:10.1002/pon.70477)
Supplement: Supplementary file 1 — Supporting Information S1 [file PON-35-e70477-s001.docx]

**A SYSTEMATIC REVIEW AND META-ANALYSIS ON THE LEVELS OF SUPPORTIVE CARE NEEDS IN ADULTS WITH CANCER**

**Appendix S1. Search strategy**

**Pubmed**

(Tumors [title/abstract] OR Neoplasia [title/abstract] OR Neoplasias [title/abstract] OR

Neoplasm [title/abstract] OR Tumor [title/abstract] OR Cancer [title/abstract] OR Cancers

[title/abstract] OR Malignancy [title/abstract] OR Carcinoma [title/abstract] OR Oncology

[title/abstract] OR Carcinogen [title/abstract]) AND (“Supportive care needs” [title/abstract]

OR “Supportive Care Need Survey – Short Form 34” [title/abstract]) NOT (Review [title])

**CINAHL (via EBSCOhost) & Web of Science**

(Tumors OR Neoplasia OR Neoplasias OR Neoplasm OR Tumor OR Cancer OR Cancers OR

Malignancy OR Carcinoma OR Oncology OR Carcinogen AND (“Supportive care needs” OR

“Supportive Care Need Survey – Short Form 34”) NOT (Review)

**EMBASE**

('malignant neoplasm':ti,ab OR 'neoplasm':ti,ab OR 'carcinoma':ti,ab OR 'carcinogen':ti,ab)

AND ('supportive care needs survey':ti,ab OR 'supportive care':ti,ab) NOT ('review':ti,ab OR

'systematic review':ti,ab OR 'meta analysis':ti,ab)

**LILACS and SciELO**

('tumores' OR 'neoplasia' OR 'neoplasias' OR 'tumor' OR 'tumores' OR 'câncer' OR 'cânceres'

OR 'malignidade' OR 'carcinoma' OR 'oncologia' OR 'carcinógeno' OR 'tumors' OR 'neoplasia'

OR 'neoplasias' OR 'neoplasm' OR 'tumor' OR 'cancer' OR 'cancers' OR 'malignancy' OR

'carcinoma' OR 'oncology' OR 'carcinogen') AND ('necessidades de cuidados de suporte' OR

'necessidades de cuidados de suporte' OR 'Encuesta de Necesidades de Cuidados de Soporte'

OR 'supportive care needs' OR 'Supportive Care Need Survey – Short Form 34')) NOT

('revisão' OR 'revisión' OR 'review')

**Table S1.** Characteristics of studies included.

| **Author** | **Participant characteristics** | **Cancer characteristics** | **Treatment characteristics** |
| --- | --- | --- | --- |
| Boyes et al. 2008 | Country: Australia  Study Design: Cross-sectional  Sample size: 414  Age: NR  Males: 43.0% | Cancer type: Mixed  Advanced stage: NR  Metastasis: NR | Surgery: NR  Radiotherapy: NR  Chemotherapy: NR  Previous hormone: NR |
| Lam et al. 2011 | Country: China & Germany  Study Design: Cross-sectional  Sample size: 640  Age: 54.5 years  Males: 0.0% | Cancer type: Breast cancer  Advanced stage: 22.7%  Metastasis: 22.7% | Surgery: 82.8%  Radiotherapy: 15.7%  Chemotherapy: 34.6%  Previous hormone: 37.7% |
| Lam et al. 2011 | Country: China  Study Design: Cross-sectional  Sample size: 348  Age: 53.7 years  Males: 0.0% | Cancer type: Breast cancer  Advanced stage: 43.4%  Metastasis: 22.7% | Surgery: 79.5%  Radiotherapy: 7.0%  Chemotherapy: 56.0%  Previous hormone: 24.0% |
| Akechi et al. 2012 | Country: Japan  Study Design: Cross-sectional  Sample size: 619  Age: 56.1 years  Males: 0.0% | Cancer type: Breast cancer  Advanced stage: 9.0%  Metastasis: 3.0% | Surgery: 8.0%  Radiotherapy: 2.0%  Chemotherapy: 17.0%  Previous hormone: 48.0% |
| Husain et al. 2012 | Country: Canada  Study Design: Cross-sectional  Sample size: 114  Age: 63.3 years  Males: 46.6% | Cancer type: Lung cancer  Advanced stage: 100.0%  Metastasis: NR | Surgery: 39.2%  Radiotherapy: 72.2%  Chemotherapy: 85.3%  Previous hormone: NR |
| Knobf et al. 2012 | Country: United States  Study Design: Cross-sectional  Sample size: 1516  Age: 61.0 years  Males: 23.6% | Cancer type: Mixed  Advanced stage: NR  Metastasis: NR | Surgery: 75.4%  Radiotherapy: 55.1%  Chemotherapy: 53.8%  Previous hormone: NR |
| Li et al. 2012 | Country: China  Study Design: Cross-sectional  Sample size: 201  Age: 59.5 years  Males: 28.9% | Cancer type: Mixed  Advanced stage: NR  Metastasis: NR | Surgery: NR  Radiotherapy: 5.5%  Chemotherapy: 28.4%  Previous hormone: NR |
| Liao et al. 2012 | Country: Taiwan  Study Design: Longitudinal  Sample size: 124  Age: 49.3 years  Males: 0.0% | Cancer type: Breast cancer  Advanced stage: 20.0%  Metastasis: 5.0% | Surgery: 100.0%  Radiotherapy: 18.0%  Chemotherapy: 77.0%  Previous hormone: NR |
| Brédart et al. 2013 | Country: France & Switzerland  Study Design: Cross-sectional  Sample size: 384  Age: 54.0 years  Males: 0.0% | Cancer type: Breast cancer  Advanced stage: NR  Metastasis: 19.3% | Surgery: 31.5%  Radiotherapy: 41.1%  Chemotherapy: 46.7%  Previous hormone: NR |
| Fielding et al. 2013 | Country: China, Taiwan & Japan  Study Design: Cross-sectional  Sample size: 552  Age: 61.8 years  Males: 60.5% | Cancer type: Colorectal cancer  Advanced stage: NR  Metastasis: NR | Surgery: 96.74%  Radiotherapy: NR  Chemotherapy: 47.6%  Previous hormone: NR |
| Henry et al. 2013 | Country: Canada  Study Design: Cross-sectional  Sample size: 127  Age: 60.6 years  Males: 66.7% | Cancer type: Head and Neck  Advanced stage: NR  Metastasis: NR | Surgery: 55.4%  Radiotherapy: 83.5%  Chemotherapy: NR  Previous hormone: NR |
| Cheng et al. 2014 | Country: Singapore  Study Design: Cross-sectional  Sample size: 150  Age: 55.1 years  Males: 0.0% | Cancer type: Breast cancer  Advanced stage: 15.3%  Metastasis: NR | Surgery: 100.0%  Radiotherapy: 70.0%  Chemotherapy: 60.6%  Previous hormone: 75.4% |
| Liao et al. 2014 | Country: Taiwan  Study Design: Longitudinal  Sample size: 80  Age: 49.3 years  Males: 0.0% | Cancer type: Breast cancer  Advanced stage: 39.0%  Metastasis: NR | Surgery: 89.0%  Radiotherapy: 10.0%  Chemotherapy: 94.0%  Previous hormone: 4.0% |
| Cheng et al. 2015 | Country: Singapore  Study Design: Cross-sectional  Sample size: 250  Age: 54.7 years  Males: 0.0% | Cancer type: Breast cancer  Advanced stage: 15.6%  Metastasis: NR | Surgery: 100.0%  Radiotherapy: 68.8%  Chemotherapy: 55.6%  Previous hormone: 80.8% |
| Galvão et al. 2015 | Country: Australia  Study Design: Cross-sectional  Sample size: 463  Age: 64.4 years  Males: 100.0% | Cancer type: Prostate cancer  Advanced stage: NR  Metastasis: NR | Surgery: 70.4%  Radiotherapy: 17.9%  Chemotherapy: NR  Previous hormone: 18.5% |
| Geue et al. 2015 | Country: Germany  Study Design: Cross-sectional  Sample size: 99  Age: 33.3 years  Males: 33.3% | Cancer type: Mixed  Advanced stage: NR  Metastasis: NR | Surgery: 61.6%  Radiotherapy: 53.5%  Chemotherapy: 82.8%  Previous hormone: NR |
| Sarkar et al. 2015 | Country: Germany  Study Design: Cross-sectional  Sample size: 335  Age: 58.7 years  Males: 63.3% | Cancer type: Mixed  Advanced stage: NR  Metastasis: NR | Surgery: NR  Radiotherapy: NR  Chemotherapy: NR  Previous hormone: NR |
| Beesley et al. 2016 | Country: Australia  Study Design: Cross-sectional  Sample size: 136  Age: 66.0 years  Males: 60.0% | Cancer type: Pancreatic cancer  Advanced stage: 47.0%  Metastasis: 34.0% | Surgery: 100.0%  Radiotherapy: NR  Chemotherapy: 82.0%  Previous hormone: NR |
| Brédart et al. 2016 | Country: France  Study Design: Longitudinal  Sample size: 278  Age: 55.3 years  Males: 0.0% | Cancer type: Breast cancer  Advanced stage: 9.4%  Metastasis: 0.0% | Surgery: 100.0%  Radiotherapy: 100.0%  Chemotherapy: 44.6%  Previous hormone: 69.06% |
| Edib et al. 2016 | Country: Malaysia  Study Design: Cross-sectional  Sample size: 117  Age: NR  Males: 0.0% | Cancer type: Breast cancer  Advanced stage: 35.9%  Metastasis: 12.0% | Surgery: 100.0%  Radiotherapy: 80.3%  Chemotherapy: 71.8%  Previous hormone: 79.3% |
| Giuliani et al. 2016 | Country: Canada  Study Design: Cross-sectional  Sample size: 89  Age: 71.0 years  Males: 49.0% | Cancer type: Lung cancer  Advanced stage: 16.0%  Metastasis: 9.0% | Surgery: NR  Radiotherapy: NR  Chemotherapy: NR  Previous hormone: NR |
| Hasegawa et al. 2016 | Country: Japan  Study Design: Cross-sectional  Sample size: 45  Age: 66.6 years  Males: 53.0% | Cancer type: Mixed  Advanced stage: NR  Metastasis: NR | Surgery: NR  Radiotherapy: NR  Chemotherapy: NR  Previous hormone: NR |
| Langbecker and Yates 2016 | Country: Australia  Study Design: Longitudinal  Sample size: 40  Age: NR  Males: 57.5% | Cancer type: Brain tumor  Advanced stage: 55.6%  Metastasis: NR | Surgery: 87.5%  Radiotherapy: 57.0%  Chemotherapy: 60.0%  Previous hormone: NR |
| Weißflog et al. 2016 | Country: Germany  Study Design: Cross-sectional  Sample size: 330  Age: 57.0 years  Males: 63.3% | Cancer type: Hematological Cancers  Advanced stage: NR  Metastasis: NR | Surgery: NR  Radiotherapy: NR  Chemotherapy: NR  Previous hormone: NR |
| Doubova et al. 2017 | Country: Mexico  Study Design: Cross-sectional  Sample size: 825  Age: 53.6 years  Males: 40.7% | Cancer type: Mixed  Advanced stage: 49.4%  Metastasis: NR | Surgery: 13.2%  Radiotherapy: NR  Chemotherapy: NR  Previous hormone: NR |
| Momino et al. 2017 | Country: Japan  Study Design: Longitudinal  Sample size: 37  Age: 55.0 years  Males: 0.0% | Cancer type: Breast Cancer  Advanced stage: 5.0%  Metastasis: NR | Surgery: 100.0%  Radiotherapy: NR  Chemotherapy: 76.0%  Previous hormone: 60.0% |
| Renovanz et al. 2017 | Country: Germany  Study Design: Cross-sectional  Sample size: 165  Age: 51.0 years  Males: 53.0% | Cancer type: Neurologic cancer  Advanced stage: 82.0%  Metastasis: NR | Surgery: 68.0%  Radiotherapy: NR  Chemotherapy: 36.0%  Previous hormone: NR |
| So et al. 2017 | Country: China  Study Design: Cross-sectional  Sample size: 285  Age: 51.8 years  Males: 75.5% | Cancer type: Head and Neck  Advanced stage: 47.2%  Metastasis: 0% | Surgery: 47.2%  Radiotherapy: 98.1%  Chemotherapy: 81.1%  Previous hormone: NR |
| Shun et al. [1] 2017 | Country: Taiwan  Study Design: Longitudinal  Sample size: 90  Age: 62.5 years  Males: 72.2% | Cancer type: Liver cancer  Advanced stage: NR  Metastasis: NR | Surgery: NR  Radiotherapy: NR  Chemotherapy: NR  Previous hormone: NR |
| Shun et al. [2] 2017 | Country: Taiwan  Study Design: Cross-sectional  Sample size: 104  Age: 54.2 years  Males: 76.4% | Cancer type: Liver cancer  Advanced stage: NR  Metastasis: NR | Surgery: NR  Radiotherapy: NR  Chemotherapy: NR  Previous hormone: NR |
| Yu et al. 2017 | Country: China  Study Design: Cross-sectional  Sample size: 311  Age: NR  Males: 59.5% | Cancer type: Leukemia  Advanced stage: NR  Metastasis: NR | Surgery: NR  Radiotherapy: NR  Chemotherapy: NR  Previous hormone: NR |
| Javadi et al. 2018 | Country: Iran  Study Design: Longitudinal  Sample size: 64  Age: NR  Males: 0.0% | Cancer type: Breast cancer  Advanced stage: NR  Metastasis: 0.0% | Surgery: 35.0%  Radiotherapy: 5.0%  Chemotherapy: 22.0%  Previous hormone: NR |
| Moreno et al. 2018 | Country: United States  Study Design: Cross-sectional  Sample size: 288  Age: 56.0 years  Males: 46.2% | Cancer type: Mixed  Advanced stage: 25.0%  Metastasis: NR | Surgery: NR  Radiotherapy: 56.6%  Chemotherapy: 47.3%  Previous hormone: 46.9% |
| Pérez-Fortis et al. 2018 | Country: Mexico  Study Design: Cross-sectional  Sample size: 155  Age: 53.0 years  Males: 0.0% | Cancer type: Breast cancer  Advanced stage: NR  Metastasis: NR | Surgery: 18.7%  Radiotherapy: NR  Chemotherapy: NR  Previous hormone: NR |
| Sleight et al. 2018 | Country: United States  Study Design: Cross-sectional  Sample size: 99  Age: 54.0 years  Males: 0.0% | Cancer type: Breast cancer  Advanced stage: 3.0%  Metastasis: 0.0% | Surgery: 13.0%  Radiotherapy: 4.0%  Chemotherapy: 1.0%  Previous hormone: NR |
| Wang et al. 2018 | Country: China  Study Design: Cross-sectional  Sample size: 264  Age: NR  Males: 0.0% | Cancer type: Breast cancer  Advanced stage: 31.4%  Metastasis: 9.8% | Surgery: 100.0%  Radiotherapy: 28.0%  Chemotherapy: 72.3%  Previous hormone: NR |
| Livingston et al. 2019 | Country: Australia  Sample size: 82  Age: 59.5 years  Males: 29.0% | Cancer type: Mixed  Advanced stage: NR  Metastasis: NR | Surgery: NR  Radiotherapy: 49.0%  Chemotherapy: 51.0%  Previous hormone: NR |
| Miniotti et al. 2019 | Country: Italy  Study Design: Cross-sectional  Sample size: 203  Age: NR  Males: 56.6% | Cancer type: Colorectal cancer  Advanced stage: 100.0%  Metastasis: 65.5% | Surgery: 89.2%  Radiotherapy: 20.2%  Chemotherapy: 100.0%  Previous hormone: NR |
| Mirzaei et al. 2019 | Country: Iran  Study Design: Cross-sectional  Sample size: 190  Age: 46.9 years  Males: 0.0% | Cancer type: Breast Cancer  Advanced stage: 38.9%  Metastasis: 5.3% | Surgery: 100.0%  Radiotherapy: 51.6%  Chemotherapy: 48.4%  Previous hormone: NR |
| Nimekari et al. 2019 | Country: Iran  Study Design: Cross-sectional  Sample size: 150  Age: 45.8 years  Males: 0.0% | Cancer type: Breast Cancer  Advanced stage: NR  Metastasis: NR | Surgery: NR  Radiotherapy: NR  Chemotherapy: NR  Previous hormone: NR |
| Rha et al. 2019 | Country: Korea  Study Design: Cross-sectional  Sample size: 223  Age: 56.0 years  Males: 59.2% | Cancer type: Gastric cancer  Advanced stage: 73.5%  Metastasis: 39.0% | Surgery: 0.0%  Radiotherapy: 0.0%  Chemotherapy: 100.0%  Previous hormone: NR |
| Sender et al. 2019 | Country: Germany  Study Design: Longitudinal  Sample size: 514  Age: 29.6 years  Males: 24.9% | Cancer type: Mixed  Advanced stage: NR  Metastasis: NR | Surgery: 73.7%  Radiotherapy: 47.1%  Chemotherapy: 76.3%  Previous hormone: NR |
| Watson et al. 2019 | Country: England  Study Design: Cross-sectional  Sample size: 212  Age: 68.0 years  Males: 100.0% | Cancer type: Prostate cancer  Advanced stage: NR  Metastasis: NR | Surgery: 36.5%  Radiotherapy: 39.7%  Chemotherapy: NR  Previous hormone: 43.8% |
| Choi et al. [1] 2020 | Country: China  Study Design: Cross-sectional  Sample size: 1102  Age: 55.4 years  Males: 40.7% | Cancer type: Mixed  Advanced stage: 37.34%  Metastasis: 7.3% | Surgery: NR  Radiotherapy: NR  Chemotherapy: NR  Previous hormone: NR |
| Choi et al. [2] 2020 | Country: South Korea  Study Design: Cross-sectional  Sample size: 118  Age: 65.2 years  Males: 92.4% | Cancer type: Esophageal cancer  Advanced stage: 21.4%  Metastasis: NR | Surgery: 100.0%  Radiotherapy: 34.5%  Chemotherapy: 99.3%  Previous hormone: NR |
| Doubova;Martinez-Vega et al. 2020 | Country: Mexico  Study Design: Cross-sectional  Sample size: 1664  Age: 55.0 years  Males: 45.01% | Cancer type: Mixed  Advanced stage: NR  Metastasis: NR | Surgery: 15.75%  Radiotherapy: 66.0%  Chemotherapy: 66.0%  Previous hormone: NR |
| Fatiregun et al. 2020 | Country: Nigeria  Study Design: Cross-sectional  Sample size: 205  Age: 47.4 years  Males: 3.4% | Cancer type: Mixed  Advanced stage: NR  Metastasis: NR | Surgery: 19.0%  Radiotherapy: 21.5%  Chemotherapy: 62.4%  Previous hormone: 7.3% |
| Graf et al. 2020 | Country: Germany  Study Design: Cross-sectional  Sample size: 771  Age: 50.6 years  Males: 0.0% | Cancer type: Breast and gynecologic cancer  Advanced stage: NR  Metastasis: 14.4% | Surgery: NR  Radiotherapy: NR  Chemotherapy: NR  Previous hormone: NR |
| Guccione et al. 2020 | Country: Australia  Study Design: Cross-sectional  Sample size: 123  Age: 60.7 years  Males: 51.0% | Cancer type: Neuroendocrine tumours  Advanced stage: 47.97%  Metastasis: 43.9% | Surgery: NR  Radiotherapy: NR  Chemotherapy: NR  Previous hormone: NR |
| Henry et al. 2020 | Country: Canada  Study Design: Longitudinal  Sample size: 145  Age: 63.3 years  Males: 69.7% | Cancer type: Head and Neck  Advanced stage: 72.5%  Metastasis: NR | Surgery: 36.2%  Radiotherapy: 85.8%  Chemotherapy: 67.1%  Previous hormone: NR |
| Jie et al. 2020 | Country: China  Study Design: Cross-sectional  Sample size: 306  Age: NR  Males: 57.8% | Cancer type: Colorectal cancer  Advanced stage: 32.5%  Metastasis: 8.7% | Surgery: 100.0%  Radiotherapy: NR  Chemotherapy: 36.8%  Previous hormone: NR |
| Lyu et al. 2020 | Country: China  Study Design: Cross-sectional  Sample size: 205  Age: 47.2 years  Males: 60.6% | Cancer type: Head and Neck  Advanced stage: NR  Metastasis: NR | Surgery: 39.8%  Radiotherapy: 26.2%  Chemotherapy: 20.39%  Previous hormone: NR |
| Paterson et al. 2020 | Country: UK & Australia  Study Design: Cross-sectional  Sample size: 102  Age: NR  Males: 100.0% | Cancer type: Prostate cancer  Advanced stage: NR  Metastasis: NR | Surgery: NR  Radiotherapy: 0.0%  Chemotherapy: 0.0%  Previous hormone: 93.1% |
| Sender et al. 2020 | Country: Germany  Study Design: Longitudinal  Sample size: 117  Age: NR  Males: 34.1% | Cancer type: Mixed  Advanced stage: NR  Metastasis: NR | Surgery: 63.3%  Radiotherapy: 59.0%  Chemotherapy: 82.9%  Previous hormone: NR |
| van der Hout et al. 2020 | Country: Netherlands  Study Design: Longitudinal  Sample size: 624  Age: 65.0  Males: 49.4% | Cancer type: Mixed  Advanced stage: 39.0%  Metastasis: 18.6% | Surgery: NR  Radiotherapy: NR  Chemotherapy: NR  Previous hormone: NR |
| Zhu et al. 2020 | Country: China  Sample size: 135  Age: 55.0 years  Males: 74.1% | Cancer type: Colorectal cancer  Advanced stage: NR  Metastasis: NR | Surgery: NR  Radiotherapy: NR  Chemotherapy: 0.0%  Previous hormone: NR |
| Al-Azri et al. 2021 | Country: Oman  Study Design: Cross-sectional  Sample size: 120  Age: NR  Males: 0.0% | Cancer type: Breast cancer  Advanced stage: 42.2%  Metastasis: NR | Surgery: 5.6%  Radiotherapy: 5.6%  Chemotherapy: 15.4%  Previous hormone: 14.1% |
| Al-Husban et al. 2021 | Country: Jordan  Study Design: Cross-sectional  Sample size: 180  Age: 54.3 years  Males: 62.2% | Cancer type: Colorectal cancer  Advanced stage: 34.4%  Metastasis: 17.2% | Surgery: 81.7%  Radiotherapy: 88.3%  Chemotherapy: 88.3%  Previous hormone: NR |
| Chee et al. 2021 | Country: China, Korea & Japan  Study Design: Longitudinall  Sample size: 99  Age: 52.5 years  Males: 0.0% | Cancer type: Breast cancer  Advanced stage: 17.2%  Metastasis: 5.4% | Surgery: 73.7%  Radiotherapy: 48.5%  Chemotherapy: 45.5%  Previous hormone: 41.4% |
| Chung and Lin 2021 | Country: China  Study Design: Cross-sectional  Sample size: 311  Age: 51.5 years  Males: 31.8% | Cancer type: Mixed  Advanced stage: NR  Metastasis: 5.8% | Surgery: NR  Radiotherapy: NR  Chemotherapy: NR  Previous hormone: NR |
| Damen et al. 2021 | Country: Netherlands  Study Design: Cross-sectional  Sample size: 122  Age: 69.2 years  Males: 56.0% | Cancer type: Haematological Cancers  Advanced stage: NR  Metastasis: NR | Surgery: NR  Radiotherapy: NR  Chemotherapy: NR  Previous hormone: NR |
| Dionisi-Vici et al. 2021 | Country: Italy  Study Design: Longitudinal  Sample size: 73  Age: 56.9 years  Males: 24.7% | Cancer type: Thyroid cancer  Advanced stage: NR  Metastasis: NR | Surgery: NR  Radiotherapy: NR  Chemotherapy: NR  Previous hormone: NR |
| Hinte et al. 2021 | Country: Netherlands  Study Design: Cross-sectional  Sample size: 50  Age: 69.0 years  Males: 56.0% | Cancer type: Head and Neck  Advanced stage: NR  Metastasis: NR | Surgery: 88.0%  Radiotherapy: 62.0%  Chemotherapy: 18.0%  Previous hormone: 0.0% |
| Husson et al. 2021 | Country: Multiple countries  Study Design: Cross-sectional  Sample size: 115  Age: 47.0 years  Males: 23.4% | Cancer type: Epithelioid Haemangioendothelioma  Advanced stage: 6.9%  Metastasis: NR | Surgery: 50.0%  Radiotherapy: 17.0%  Chemotherapy: 54.0%  Previous hormone: NR |
| Krug et al. 2021 | Country: Germany  Study Design: Longitudinal  Sample size: 154  Age: 66.3 years  Males: 55.4% | Cancer type: Lung cancer  Advanced stage: 100.0%  Metastasis: 100.0% | Surgery: NR  Radiotherapy: NR  Chemotherapy: NR  Previous hormone: NR |
| Lee et al. 2021 | Country: Taiwan  Study Design: Cross-sectional  Sample size: 234  Age: 71.8 years  Males: 55.7% | Cancer type: Mixed  Advanced stage: 62.9%  Metastasis: NR | Surgery: NR  Radiotherapy: NR  Chemotherapy: NR  Previous hormone: NR |
| Yuce et al. 2021 | Country: Turkey  Study Design: Cross-sectional  Sample size: 298  Age: 57.1 years  Males: 50.0% | Cancer type: Mixed  Advanced stage: NR  Metastasis: 54.4% | Surgery: NR  Radiotherapy: NR  Chemotherapy: 71.5%  Previous hormone: NR |
| Abu-Odah et al. 2022 | Country: Palestine  Study Design: Cross-sectional  Sample size: 379  Age: 50.1 years  Males: 50.9% | Cancer type: Mixed  Advanced stage: 100.0%  Metastasis: 50.9% | Surgery: 4.2%  Radiotherapy: 7.1%  Chemotherapy: 81.0%  Previous hormone: NR |
| Al-Omari et al. 2022 | Country: Jordan  Study Design: Cross-sectional  Sample size: 240  Age: NR  Males: 30.4% | Cancer type: Mixed  Advanced stage: NR  Metastasis: NR | Surgery: NR  Radiotherapy: NR  Chemotherapy: NR  Previous hormone: NR |
| Benedict et al. 2022 | Country: United States  Study Design: Cross-sectional  Sample size: 273  Age: 54.7 years  Males: 0.0% | Cancer type: Breast and gynecologic cancer  Advanced stage: 33.0%  Metastasis: 11.4% | Surgery: 91.5%  Radiotherapy: 54.6%  Chemotherapy: 72.5%  Previous hormone: 100.0% |
| Calvo-Schimmel et al. 2022 | Country: United States  Study Design: Cross-sectional  Sample size: 188  Age: 69.0 years  Males: 100.0% | Cancer type: Prostate cancer  Advanced stage: 77.9%  Metastasis: 49.7% | Surgery: 60.1%  Radiotherapy: 63.3%  Chemotherapy: 22.3%  Previous hormone: NR |
| Ebrahimabadi et al. 2022 | Country: Iran  Study Design: Longitudinal  Sample size: 60  Age: 51.2 years  Males: 56.6% | Cancer type: Mixed  Advanced stage: NR  Metastasis: 0.0% | Surgery: NR  Radiotherapy: NR  Chemotherapy: 23.3%  Previous hormone: NR |
| Eggins et al. 2022 | Country: Australia  Study Design: Cross-sectional  Sample size: 2635  Age: NR  Males: 0.0% | Cancer type: Breast cancer  Advanced stage: NR  Metastasis: NR | Surgery: NR  Radiotherapy: NR  Chemotherapy: NR  Previous hormone: NR |
| Jaafar et al. 2022 | Country: Malaysia  Study Design: Cross-sectional  Sample size: 190  Age: 52.8 years  Males: 54.2% | Cancer type: Head and Neck  Advanced stage: 51.0%  Metastasis: 18.4% | Surgery: 50.5%  Radiotherapy: 70.5%  Chemotherapy: 83.7%  Previous hormone: NR |
| Cai and Gou 2023 | Country: China  Study Design: Cross-sectional  Sample size: 102  Age: NR  Males: 26.5% | Cancer type: Thyroid cancer  Advanced stage: NR  Metastasis: NR | Surgery: 100.0%  Radiotherapy: NR  Chemotherapy: NR  Previous hormone: NR |
| Dhakal et al. 2023 | Country: Nepal  Study Design: Cross-sectional  Sample size: 218  Age: 53.5 years  Males: 0.0% | Cancer type: Cervical cancer  Advanced stage: 39.0%  Metastasis: 6.4% | Surgery: 17.6%  Radiotherapy: 96.0%  Chemotherapy: 81.3%  Previous hormone: NR |
| Im et al. 2023 | Country:  Study Design: Cross-sectional  Sample size: 135  Age: 52.7 years  Males: 0.0% | Cancer type: Breast cancer  Advanced stage: 13.5%  Metastasis: 4.5% | Surgery: NR  Radiotherapy: NR  Chemotherapy: NR  Previous hormone: NR |
| Masoudi et al. 2023 | Country:  Study Design: Cross-sectional  Sample size: 247  Age: 46.1 years  Males: 45.3% | Cancer type: Mixed  Advanced stage: NR  Metastasis: NR | Surgery: NR  Radiotherapy: NR  Chemotherapy: 96.0%  Previous hormone: NR |
| Molenaar et al. 2023 | Country: Netherlands  Study Design: Longitudinal  Sample size: 559  Age: 63.0 years  Males: 74.1% | Cancer type: Head and Neck  Advanced stage: 57.8%  Metastasis: 41.6% | Surgery: 38.4%  Radiotherapy: 79.6%  Chemotherapy: 31.1%  Previous hormone: NR |
| Thompson et al. 2023 | Country: Australia  Study Design: Cross-sectional  Sample size: 156  Age: 57.9 years  Males: 33.7% | Cancer type: Skin cancer  Advanced stage: 64.1%  Metastasis: NR | Surgery: 78.2%  Radiotherapy: 19.87%  Chemotherapy: 2.5%  Previous hormone: NR |
| Xiangting et al. 2023 | Country: China  Study Design: Cross-sectional  Sample size: 175  Age: 56.1 years  Males: 58.9% | Cancer type: Colorectal cancer  Advanced stage: 75.4%  Metastasis: 17.7% | Surgery: 100.0%  Radiotherapy: NR  Chemotherapy: 69.1%  Previous hormone: NR |
| Al-Qadire et al. 2024 | Country: Oman  Study Design: Cross-sectional  Sample size: 551  Age: 45.8 years  Males: 34.5% | Cancer type: Mixed  Advanced stage: 36.3%  Metastasis: NR | Surgery: 46.5%  Radiotherapy: NR  Chemotherapy: 92.2%  Previous hormone: NR |
| DiSipio et al. 2024 | Country: Australia  Study Design: Longitudinal  Sample size: 288  Age: 60.0 years  Males: 0.0% | Cancer type: Ovarian cancer  Advanced stage: 92.0%  Metastasis: NR | Surgery: NR  Radiotherapy: NR  Chemotherapy: NR  Previous hormone: NR |
| Esquives et al. 2024 | Country: United States Study Design: Cross-sectional  Sample size: 288  Age: 56.1 years  Males: 46.1% | Cancer type: Mixed  Advanced stage: 25.0%  Metastasis: 0.0% | Surgery: 88.0%  Radiotherapy: 60.1%  Chemotherapy: 54.1%  Previous hormone: 51.5% |
| Hu et al. 2024 | Country: China  Study Design: Cross-sectional  Sample size: 140  Age: NR  Males: 57.9% | Cancer type: Rectal cancer  Advanced stage: NR  Metastasis: NR | Surgery: 100.0%  Radiotherapy: NR  Chemotherapy: NR  Previous hormone: NR |
| Sánchez et al. 2024 | Country: Mexico  Study Design: Longitudinal  Sample size: 50  Age: 53.3 years  Males: 0.0% | Cancer type: Breast cancer  Advanced stage: 44.0%  Metastasis: 0.0% | Surgery: NR  Radiotherapy: 50.0%  Chemotherapy: 50.0%  Previous hormone: NR |
| Temiz et al. 2024 | Country: Turkey  Study Design: Longitudinal  Sample size: 28  Age: 55.3 years  Males: 0.0% | Cancer type: Breast cancer  Advanced stage: 10.7%  Metastasis: NR | Surgery: NR  Radiotherapy: 100.0%  Chemotherapy: NR  Previous hormone: NR |
| Arasu et al. 2025 | Country: Australia  Study Design: Cross-sectional  Sample size: 3323  Age: NR  Males: 0.0% | Cancer type: Breast cancer  Advanced stage: NR  Metastasis: NR | Surgery: NR  Radiotherapy: NR  Chemotherapy: NR  Previous hormone: NR |
| Chen et al. 2025 | Country: China  Study Design: Cross-sectional  Sample size: 486  Age: NR  Males: 61.7% | Cancer type: Lung cancer  Advanced stage: 76.3%  Metastasis: 54.7% | Surgery: 16.1%  Radiotherapy: 4.5%  Chemotherapy: 90.7%  Previous hormone: NR |
| Park et al. 2025 | Country: South Korea  Study Design: Cross-sectional  Sample size: 208  Age: 54.6 years  Males: 51.0% | Cancer type: Mixed  Advanced stage: NR  Metastasis: NR | Surgery: 58.1%  Radiotherapy: 44.2%  Chemotherapy: 68.7%  Previous hormone: NR |
| Tang et al. 2025 | Country: China  Study Design: Cross-sectional  Sample size: 160  Age: NR  Males: 0.0% | Cancer type: Breast cancer  Advanced stage: 46.25%  Metastasis: 3.75% | Surgery: 100.0%  Radiotherapy: NR  Chemotherapy: 100.0%  Previous hormone: NR |
| Wang et al. 2025 | Country: China  Study Design: Cross-sectional  Sample size: 137  Age: 57.5 years  Males: 51.1% | Cancer type: Mixed  Advanced stage: NR  Metastasis: NR | Surgery: NR  Radiotherapy: NR  Chemotherapy: NR  Previous hormone: NR |
| Xiao et al. [1] 2025 | Country: New Zealand  Study Design: Cross-sectional  Sample size: 1075  Age: 66.5 years  Males: 100.0% | Cancer type: Prostate cancer  Advanced stage: NR  Metastasis: NR | Surgery: NR  Radiotherapy: NR  Chemotherapy: NR  Previous hormone: NR |
| Xiao et al. [2] 2025 | Country: New Zealand  Study Design: Cross-sectional  Sample size: 891  Age: NR  Males: 100.0% | Cancer type: Prostate cancer  Advanced stage: NR  Metastasis: NR | Surgery: 44.9%  Radiotherapy: 34.5%  Chemotherapy: NR  Previous hormone: 22.4% |
| Yeernuer et al. 2025 | Country: China  Study Design: Longitudinal  Sample size: 122  Age: NR  Males: 46.7% | Cancer type: Cellar cancer  Advanced stage: NR  Metastasis: NR | Surgery: NR  Radiotherapy: NR  Chemotherapy: NR  Previous hormone: NR |

NR, not reported.

**Table S2.** Overall mean scores (95% CI) for the Supportive Care Needs Survey–Short Form 34 domains using the questionnaire-based sum scores.

|  | **k** | **Mean** | **95% CI** | **p-value** | **Q** | **I^2^** | $\hat{\tau}^{2}$ | **p-value** |
| --- | --- | --- | --- | --- | --- | --- | --- | --- |
| **Overall** |  |  |  |  |  |  |  |  |
| **Health system and information (8-40)** | 17 | 27.1 | 23.9 to 30.3 | <0.001 | 2594.7 | 99.4% | 45.2 | <0.001 |
| **Psychological (10-50)** | 18 | 24.3 | 21.2 to 27.3 | <0.001 | 2396.2 | 99.3% | 43.4 | <0.001 |
| **Physical and daily living (5-25)** | 18 | 11.8 | 10.3 to 13.4 | <0.001 | 2661.0 | 99.4% | 11.6 | <0.001 |
| **Patient care and support (5-25)** | 18 | 10.8 | 9.5 to 12.1 | <0.001 | 3598.1 | 99.5% | 7.7 | <0.001 |
| **Sexuality (3-15)** | 18 | 5.5 | 4.6 to 6.4 | <0.001 | 1974.8 | 99.1% | 3.6 | <0.001 |

**Table S3.** Meta-regression analysis on the association of age, males, stage III-IV, metastasis, surgery, radiotherapy, chemotherapy and previous hormone with mean scores for the health system and information domain.

|  | **k** | **Estimate** | **SE** | **p-value** |
| --- | --- | --- | --- | --- |
| **Age, years** | 56 | -0.750 | 0.281 | 0.008 |
| **Males, %** | 70 | -0.096 | 0.065 | 0.140 |
| **Stage III-IV, %** | 40 | -0.088 | 0.089 | 0.323 |
| **Metastasis, %** | 28 | 0.012 | 0.102 | 0.907 |
| **Surgery, %** | 44 | -0.056 | 0.067 | 0.405 |
| **Radiotherapy, %** | 39 | -0.119 | 0.092 | 0.193 |
| **Chemotherapy, %** | 45 | 0.043 | 0.090 | 0.635 |
| **Previous hormone, %** | 14 | -0.057 | 0.171 | 0.739 |

**Table S4.** Meta-regression analysis on the association of age, males, stage III-IV, metastasis, surgery, radiotherapy, chemotherapy and previous hormone with mean scores for the psychological domain.

|  | **k** | **Estimate** | **SE** | **p-value** |
| --- | --- | --- | --- | --- |
| **Age, years** | 56 | -0.600 | 0.261 | 0.022 |
| **Males, %** | 70 | -0.050 | 0.059 | 0.401 |
| **Stage III-IV, %** | 40 | 0.013 | 0.085 | 0.874 |
| **Metastasis, %** | 28 | 0.028 | 0.118 | 0.814 |
| **Surgery, %** | 44 | -0.077 | 0.063 | 0.224 |
| **Radiotherapy, %** | 39 | 0.020 | 0.090 | 0.828 |
| **Chemotherapy, %** | 45 | 0.059 | 0.093 | 0.528 |
| **Previous hormone, %** | 14 | 0.078 | 0.177 | 0.659 |

**Table S5.** Meta-regression analysis on the association of age, males, stage III-IV, metastasis, surgery, radiotherapy, chemotherapy and previous hormone with mean scores for the physical and daily living domain.

|  | **k** | **Estimate** | **SE** | **p-value** |
| --- | --- | --- | --- | --- |
| **Age, years** | 57 | -0.454 | 0.265 | 0.086 |
| **Males, %** | 71 | 0.001 | 0.061 | 0.985 |
| **Stage III-IV, %** | 40 | 0.052 | 0.082 | 0.520 |
| **Metastasis, %** | 28 | 0.116 | 0.107 | 0.279 |
| **Surgery, %** | 45 | -0.146 | 0.057 | 0.011 |
| **Radiotherapy, %** | 40 | 0.016 | 0.089 | 0.859 |
| **Chemotherapy, %** | 46 | 0.088 | 0.092 | 0.338 |
| **Previous hormone, %** | 15 | 0.077 | 0.156 | 0.622 |

**Table S6.** Meta-regression analysis on the association of age, males, stage III-IV, metastasis, surgery, radiotherapy, chemotherapy and previous hormone with mean scores for the patient care and support domain.

|  | **k** | **Estimate** | **SE** | **p-value** |
| --- | --- | --- | --- | --- |
| **Age, years** | 52 | -0.756 | 0.288 | 0.009 |
| **Males, %** | 66 | -0.049 | 0.066 | 0.459 |
| **Stage III-IV, %** | 37 | -0.084 | 0.077 | 0.280 |
| **Metastasis, %** | 25 | -0.009 | 0.094 | 0.921 |
| **Surgery, %** | 42 | -0.118 | 0.059 | 0.045 |
| **Radiotherapy, %** | 37 | -0.076 | 0.097 | 0.436 |
| **Chemotherapy, %** | 43 | -0.007 | 0.097 | 0.941 |
| **Previous hormone, %** | 13 | 0.097 | 0.196 | 0.620 |

**Table S7.** Meta-regression analysis on the association of age, males, stage III-IV, metastasis, surgery, radiotherapy, chemotherapy and previous hormone with mean scores for the sexuality domain.

|  | **k** | **Estimate** | **SE** | **p-value** |
| --- | --- | --- | --- | --- |
| **Age, years** | 57 | -0.390 | 0.228 | 0.087 |
| **Males, %** | 71 | 0.009 | 0.056 | 0.879 |
| **Stage III-IV, %** | 40 | -0.012 | 0.070 | 0.861 |
| **Metastasis, %** | 28 | 0.046 | 0.107 | 0.668 |
| **Surgery, %** | 45 | -0.114 | 0.051 | 0.025 |
| **Radiotherapy, %** | 40 | 0.007 | 0.080 | 0.934 |
| **Chemotherapy, %** | 46 | -0.045 | 0.090 | 0.623 |
| **Previous hormone, %** | 14 | 0.100 | 0.183 | 0.583 |

**Table S8.** Meta-regression analysis on the association of age, males, stage III-IV, metastasis, surgery, radiotherapy, chemotherapy and previous hormone with mean scores for the total score domain.

|  | **k** | **Estimate** | **SE** | **p-value** |
| --- | --- | --- | --- | --- |
| **Age, years** | 16 | -1.341 | 0.770 | 0.082 |
| **Males, %** | 16 | 0.007 | 0.155 | 0.966 |
| **Stage III-IV, %** | 9 | - | - | - |
| **Metastasis, %** | 5 | - | - | - |
| **Surgery, %** | 8 | - | - | - |
| **Radiotherapy, %** | 6 | - | - | - |
| **Chemotherapy, %** | 11 | 0.254 | 0.188 | 0.178 |
| **Previous hormone, %** | 3 | - | - | - |


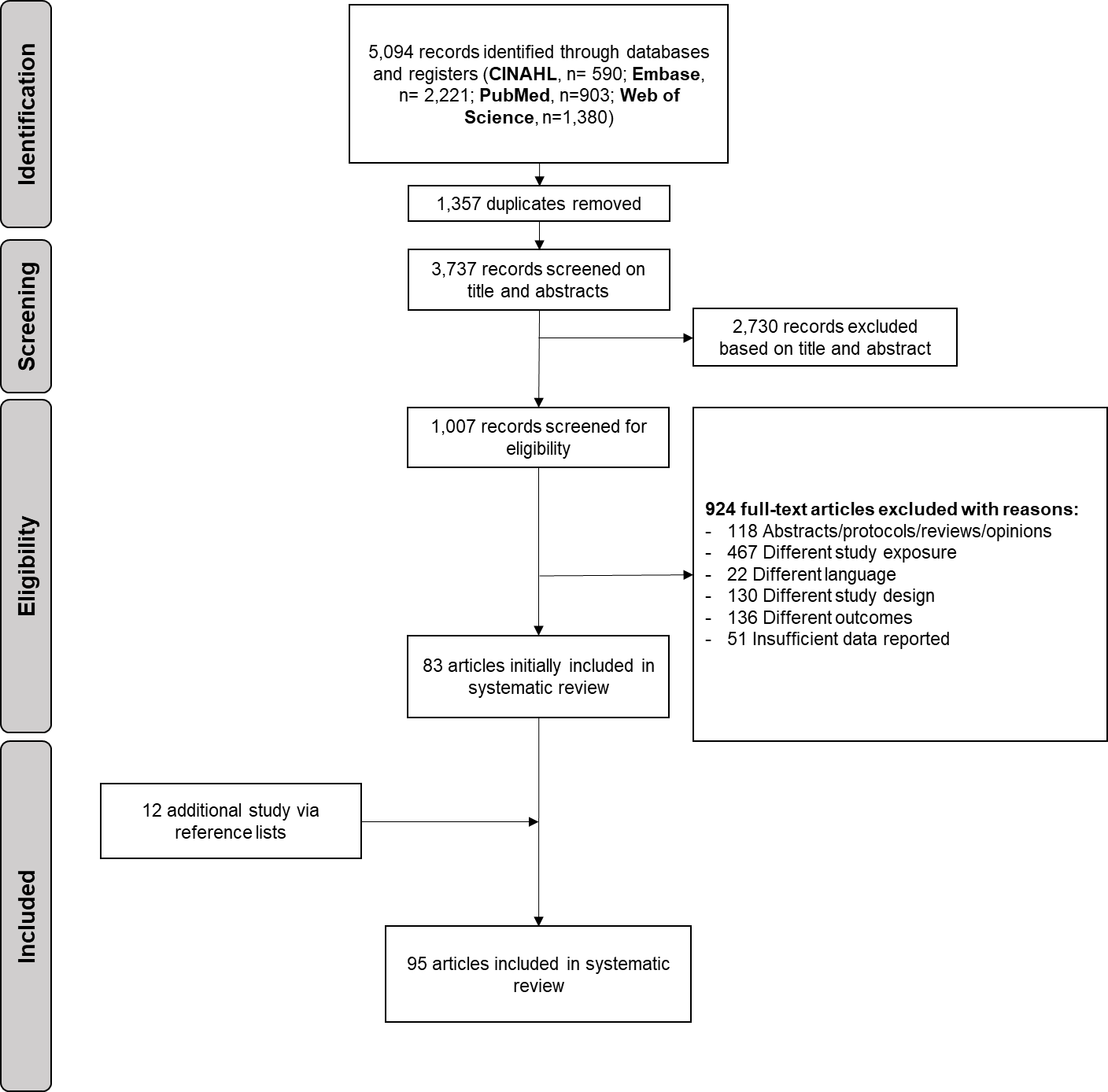


**Figure S1.** Study selection flowchart.


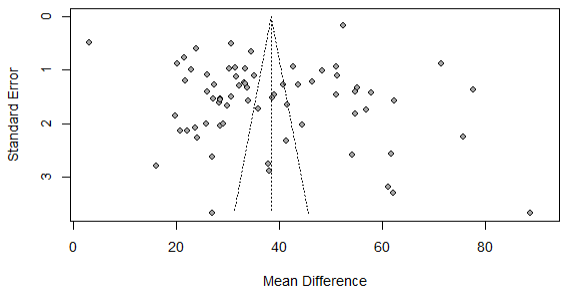


**Figure S2.** Funnel plot for the health system and information domain.


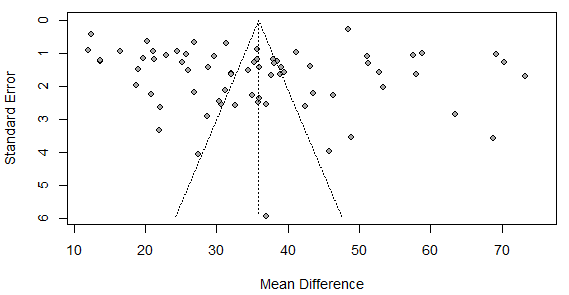


**Figure S3.** Funnel plot for the psychological domain.


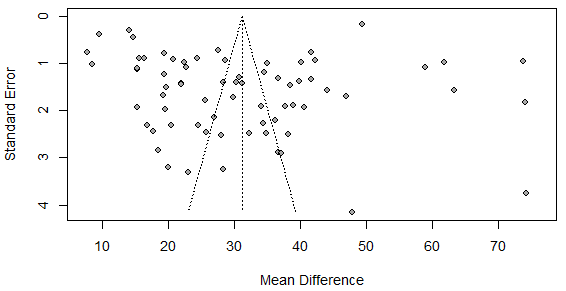


**Figure S4.** Funnel plot for the physical and daily living domain.


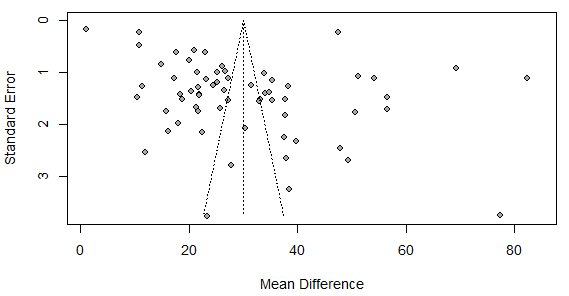


**Figure S5.** Funnel plot for the patient care and support domain.


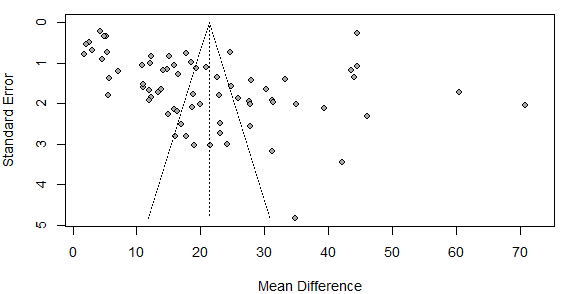


**Figure S6.** Funnel plot for the sexuality domain.


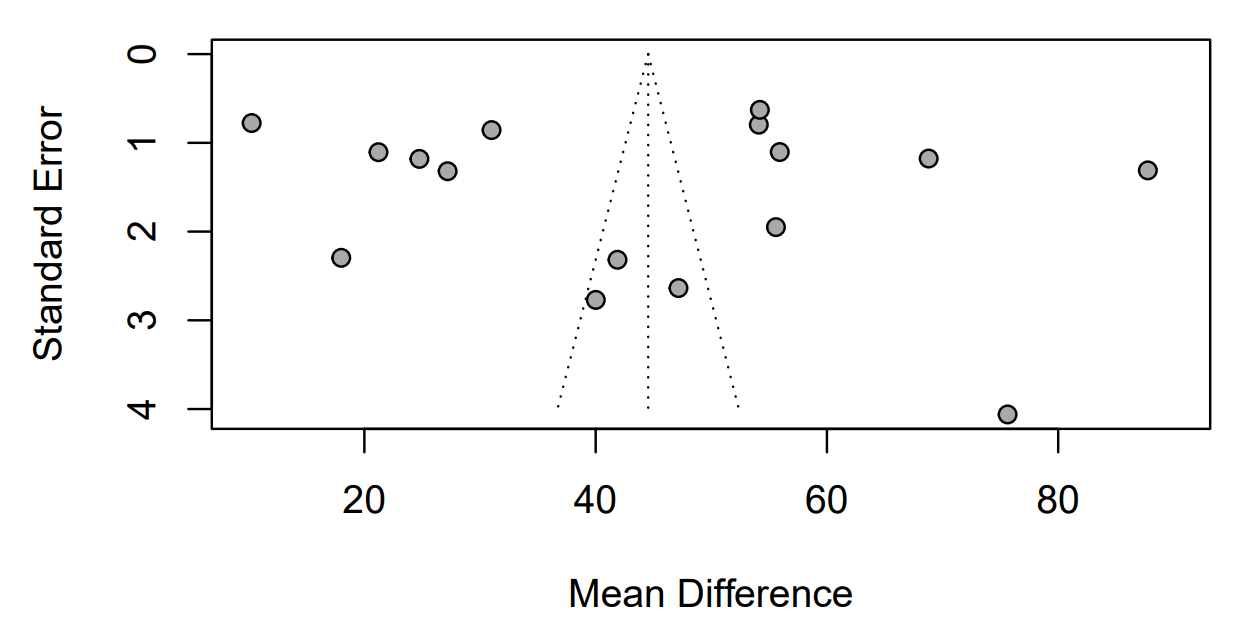


**Figure S7.** Funnel plot for the total score.
